# Grape Cultivar and Sap Culture Conditions Affect the Development of *Xylella fastidiosa* Phenotypes Associated with Pierce's Disease

**DOI:** 10.1371/journal.pone.0160978

**Published:** 2016-08-10

**Authors:** Lingyun Hao, Paulo A. Zaini, Harvey C. Hoch, Thomas J. Burr, Patricia Mowery

**Affiliations:** 1 Section of Plant Pathology and Plant-Microbe Biology, SIPS, Cornell University-New York State Agricultural Experiment Station, Geneva, New York, United States of America; 2 Department of Biology, Hobart and William Smith Colleges, Geneva, New York, United States of America; University of the West of England, UNITED KINGDOM

## Abstract

*Xylella fastidiosa* is a xylem-limited bacterium in plant hosts and causes Pierce’s disease (PD) of grapevines, which differ in susceptibility according to the *Vitis* species (spp.). In this work we compared *X*. *fastidiosa* biofilm formation and population dynamics when cultured in xylem saps from PD-susceptible and -resistant *Vitis* spp. under different conditions. Behaviors in a closed-culture system were compared to those in different sap-renewal cultures that would more closely mimic the physicochemical environment encountered *in planta*. Significant differences in biofilm formation and growth in saps from PD-susceptible and -resistant spp. were only observed using sap renewal culture. Compared to saps from susceptible *V*. *vinifera*, those from PD-resistant *V*. *aestivalis* supported lower titers of *X*. *fastidiosa* and less biofilm and *V*. *champinii* suppressed both growth and biofilm formation, behaviors which are correlated with disease susceptibility. Furthermore, in microfluidic chambers *X*. *fastidiosa* formed thick mature biofilm with three-dimensional (3-D) structures, such as pillars and mounds, in saps from all susceptible spp. In contrast, only small aggregates of various shapes were formed in saps from four out of five of the resistant spp.; sap from the resistant spp. *V*. *mustangensis* was an exception in that it also supported thick lawns of biofilm but not the above described 3-D structures typically seen in a mature biofilm from the susceptible saps. Our findings provide not only critical technical information for future bioassays, but also suggest further understanding of PD susceptibility.

## Introduction

*Xylella fastidiosa* is a xylem-limited, Gram-negative bacterium that causes devastating diseases such as Pierce’s disease (PD) in grapevines, citrus variegated chlorosis in sweet oranges, and numerous other diseases in economically important plants, including the recently identified leaf scorch and dieback disease in olive trees in Italy [1, 2]. PD symptoms are thought to be associated with secretion of effector proteins by *X*. *fastidiosa* [3–5] and/or with biofilm formation that blocks the transpiration stream flow within the host’s xylem-vascular system [6].

A number of *Vitis* species (e.g., *V*. *vinifera*, *V*. *riparia*, and *V*. *labrusca*) are particularly susceptible to PD, whereas others are resistant or tolerant (e.g. *V*. *arizonica*, *V*. *cinerea*, *V*. *champinii*, *V*. *mustangensis*, and *V*. *aestivalis*) [7–13]. *In planta* studies have demonstrated that *X*. *fastidiosa* populations are lower and biofilm is reduced in xylem vessels of certain PD-resistant *Vitis* spp. [10, 14, 15]. In addition, *X*. *fastidiosa* mutants with altered biofilm production have been reported to produce modified disease [16–23]. For example, disruption of genes involved in exopolysaccharide production in *X*. *fastidiosa* reduces adhesion to surfaces and mature biofilm formation and results in loss of virulence [20]; mutation in XatA, an autotransporter localized in cell outer membranes and membrane vesicles, impairs cell-to-cell auto-aggregation and biofilm formation and leads to reduced virulence [19]; interference of the cell signaling systems, such as production of diffusible signal factors and the secondary messenger c-di-GMP, impacts multi-cellular behaviors, including biofilm formation, and results in altered virulence [22, 23]. Although the importance of *X*. *fastidiosa* biofilm formation in disease development is well recognized, if and how the pathogen responds to plant signals in susceptible and resistant hosts and subsequently modifies its virulence behaviors, such as biofilm formation, remains unknown. Xylem saps from different grape species have been shown to impact *X*. *fastidiosa* growth, aggregation, and biofilm formation [24–26]; however, correlation between observed phenotypes and PD-susceptibility in sap is limited. Identifying factors in grape that are associated with resistance will be important for screening of grape germplasm and in the development of rational PD control strategies. Such strategies may also contribute to management of PD and other *X*. *fastidiosa*-caused diseases.

*X*. *fastidiosa* behaviors are often studied in closed culture systems, which may influence observed phenotypes due to nutrient taxing, particularly when conducted in nutrient-poor media such as xylem sap. Under such conditions the observed behaviors may differ from those that occur in the plant xylem vessels where sap is essentially consistently renewed. We therefore evaluated *X*. *fastidiosa* phenotypes in sap culture that was systematically renewed and found that *X*. *fastidiosa* behaviors differed from those in closed (non-renewed) culture. We also identified phenotype differences when *X*. *fastidiosa* was cultured in saps from PD-susceptible and -resistant spp.; more biofilm formation was supported in susceptible saps than resistant ones, which correlates with *in planta* findings [10, 14, 15]. Therefore, we propose that when studying *X*. *fastidiosa* in xylem sap, renewal is necessary to better assess bacterial behaviors. We also found that saps from PD-resistant *V*. *champinii* and *V*. *aestivalis* supported less *X*. *fastidiosa* biofilm development than those from *V*. *vinifera*, which correlated to growth differences in those saps.

## Materials and Methods

### Collection and storage of xylem sap

Grapevine xylem fluid was collected in early spring from bleeding *V*. *vinifera* Chardonnay, *V*. *riparia*, *V*. *aestivalis* and *V*. *champinii* vines at the USDA, Plant Genetic Resources Unit, Geneva, NY. Sap collection and storage procedures were as previously reported [27]. Additional sap from *V*. *vinifera* Chardonnay and *V*. *arizonica* vines was provided by Dr. Andrew Walker (University of California Davis, Davis, California), as well as from *V*. *mustangensis* and *V*. *cinerea* var. *helleri* vines provided by Dr. Mark Black (Texas A&M AgriLife Extension Services, Uvalde, Texas). *V*. *vinifera* sap from New York was used unless otherwise indicated. Sap collections from the same species and location were pooled, filtered-sterilized with a 0.2 *μ*m pore-membrane (Fisher Scientific), aliquoted and stored at -80°C. Aliquots were again filter-sterilized prior to use.

### Bacterial strains

Wild-type *X*. *fastidiosa* strain Temecula 1 (TM1) was maintained at 28°C on PW (Periwinkle Wilt) agar [28] modified by omitting phenol red, and by adding 10 mL L^-1^ of bovine serum albumin (BSA) fraction V solution (Life Technologies). The TM1 enhanced green fluorescent protein (eGFP)-tagged strain KLN59.2 [29] was cultured similarly but with the addition of 50 *μ*g mL^-1^ kanamycin. The bacteria were stored at -80°C in modified PW broth [28] containing 20% glycerol.

### Biofilm quantification in closed cultures in 96-well plates

Initial experiments were done to determine if sap from different *Vitis* spp. affected biofilm formation by *X*. *fastidiosa*. Cells from seven to 10-day-old *X*. *fastidiosa* cultures were collected from PW plates, acclimated to *V*. *vinifera* sap by serial daily passages in 50:50, 80:20, 90:10 and finally 100% sap:PD2 broth (vol:vol) as previously described [27], starting with a suspension OD_600_ = 0.05. In each passage cells were pelleted by 10,000 x g centrifugation for five minutes, and resuspended in the next sap:PD2 broth mixtures. When in 100% sap, cells were resuspended in saps from the species listed above and adjusted to an OD_600nm_ = 0.05. One hundred and fifty microliters of each sap-*X*. *fastidiosa* suspension was added to each well of 96-well polystyrene plates (Corning). The plates were then sealed with a Microseal ‘B’ adhesive seal (BioRad) before placing the lid and sealing the whole plate with Saran Wrap to prevent evaporation. Cultures were maintained for 10 days at 28°C with 185 rpm rotation. Plates were then carefully opened to prevent disruption of biofilms on well surfaces and rinsed three times with 200 *μ*L per well of distilled water to remove planktonic cells and any cells loosely attached or deposited on the bottom of the well. To measure the degree of biofilm formed by remaining attached cells, 200 *μ*L of aqueous 0.1% crystal violet was added to each well and plates were kept at room temperature (~23°C) for 20 mins. Plates were washed three times as before with water, followed by the addition of 200 μL of 1% sodium dodecyl sulfate (SDS) and agitated for five minutes. The SDS-dye solution was measured at OD_600nm_ in a Synergy 2 plate reader (Biotek). A minimum of three biological replicates was assayed for each media condition (with a minimum of three technical replicates each).

### Biofilm and population determination in semi-renewed cultures in glass tubes

To help reduce the effect of nutrient depletion on biofilm formation in closed cultures, experiments using sap renewal were conducted with saps from *V*. *vinifera* (PD susceptible), *V*. *aestivalis* (resistant) and *V*. *champinii* spp (resistant) as described above with some modifications. Briefly, *X*. *fastidiosa* cells were acclimated to each sap separately, allowing 24 h incubation for each passage: 50:50, 80:20, 90:10 100% sap:PD2 broth (vol:vol), before being inoculated into 100% sap of the same species in glass tubes. Each glass tube (13 x 100 mm) contained 2 mL of the sap-*X*. *fastidiosa* culture at an initial OD_600_ = 0.05, and was incubated at 28°C with shaking at 185 rpm. For the biofilm assays, saps were changed carefully at two, four and six-days post-inoculation without disturbing the biofilm rings: at each time point, each culture was transferred with a 5 mL pipette tip into a sterile 2.2 mL microcentrifuge tube for centrifugation at 10,000 x g for 5 mins, the supernatant was discarded and the pelleted cells were suspended in 2 mL of fresh sap of the same species in the original tube. The biofilm was quantified by crystal violet staining at day eight as previously described [27]. A closed culture control for each sap was performed as described above except the sap was not renewed. Four biological replicates from individually acclimated cells were included for each treatment and the experiment was repeated twice.

To measure bacterial populations in the culture, saps were changed the same way as described above. That is every two days bacterial cells (both planktonic and biofilm) in each glass tube were collected and centrifuged, followed by discarding the supernatant and adding fresh sap to the pelleted cells. Cells in each tube were then dispersed by extensive repetitive pipetting before immediately serially diluted and plated onto PWG (Periwinkle Wilt Gelrite) plates at days zero, four, and eight. Dispersal of cells was essential to break up aggregates as thoroughly as possible so that individual colonies on plates could be generated from single cells. The plates were incubated at 28°C for approximately 14 days and bacterial colonies were counted. Representative colonies were confirmed by PCR with *X*. *fastidiosa* specific RST31/33 primers [30]. Four replicates were included for each treatment and the experiment was repeated twice.

### Biofilm formation, aggregation, and cell viability in microfluidic chambers

Microfluidic chambers were fabricated as previously described [31]. The device used in this study had two channels 26 mm long, 1 mm wide, and 0.16 mm in height. Continuous sap flow through the chamber was maintained at 0.2 *μ*L min^-1^ with a syringe pump (PicoPlus, Harvard Apparatus), corresponding to a flow speed of ~1.250 *μ*m min^-1^. Cells were acclimated to *V*. *vinifera* sap as described in the biofilm assays in 96-well plates, and approximately 100 *μ*L of cells at OD_600nm_ = 0.05 were introduced in each channel and observed for up to 10 days. Phenotypes were evaluated in each sap type at least three times.

To determine the presence of live and dead cells, KLN59.2 cells [29] were analyzed with an Olympus BX61 confocal laser scanning microscope coupled to a 544 nm helium neon laser and a 488 nm argon laser. Images were captured and analyzed with Fluoview 5.0 software (Olympus). Non-viable (dead) cells were detected with propidium iodide (PI) (Life Technologies). After four days of culturing with continuous flow in the microfluidic chamber as described above, the syringe containing sap (*V*. *vinifera* or *V*. *champinii*) was filled with the same sap type plus PI at a final concentration of 2 *μ*g mL^-1^ and continuous flow was resumed. After an additional six days (at day 10 from the beginning of the experiment) images were captured. Live KLN59.2 cells were detected through fluorescence of the eGFP and dead cells were detected through fluorescence of PI. In merged images dead cells devoid of eGFP appear only red (as in the *V*. *champinii* samples) whereas dead cells that still retained eGFP fluorescence appear yellow (as some in the *V*. *vinifera* samples). The experiment was performed with sap from each *Vitis* spp. at least three times. Scanning electron microscopy was performed as previously described [31].

### Statistical analysis

All statistical analyses were performed using the R program. To compare the mean biofilms or populations between treatments, a linear mixed effects model was used to include fixed effects of sap type, culturing, and an interaction between sap type and culturing and random effects to capture variability between the experiments. Post hoc comparisons between the treatments were performed using Tukey’s HSD (honest significance difference) to correct for multiple comparisons. A residual analysis was performed to assess the validity of the assumptions of normality and homogeneous variances. A *p* value less than 0.01 was considered to be significant.

## Results

### Impact of sap renewal on *X*. *fastidiosa* biofilm formation in different saps

**Fig 1:**
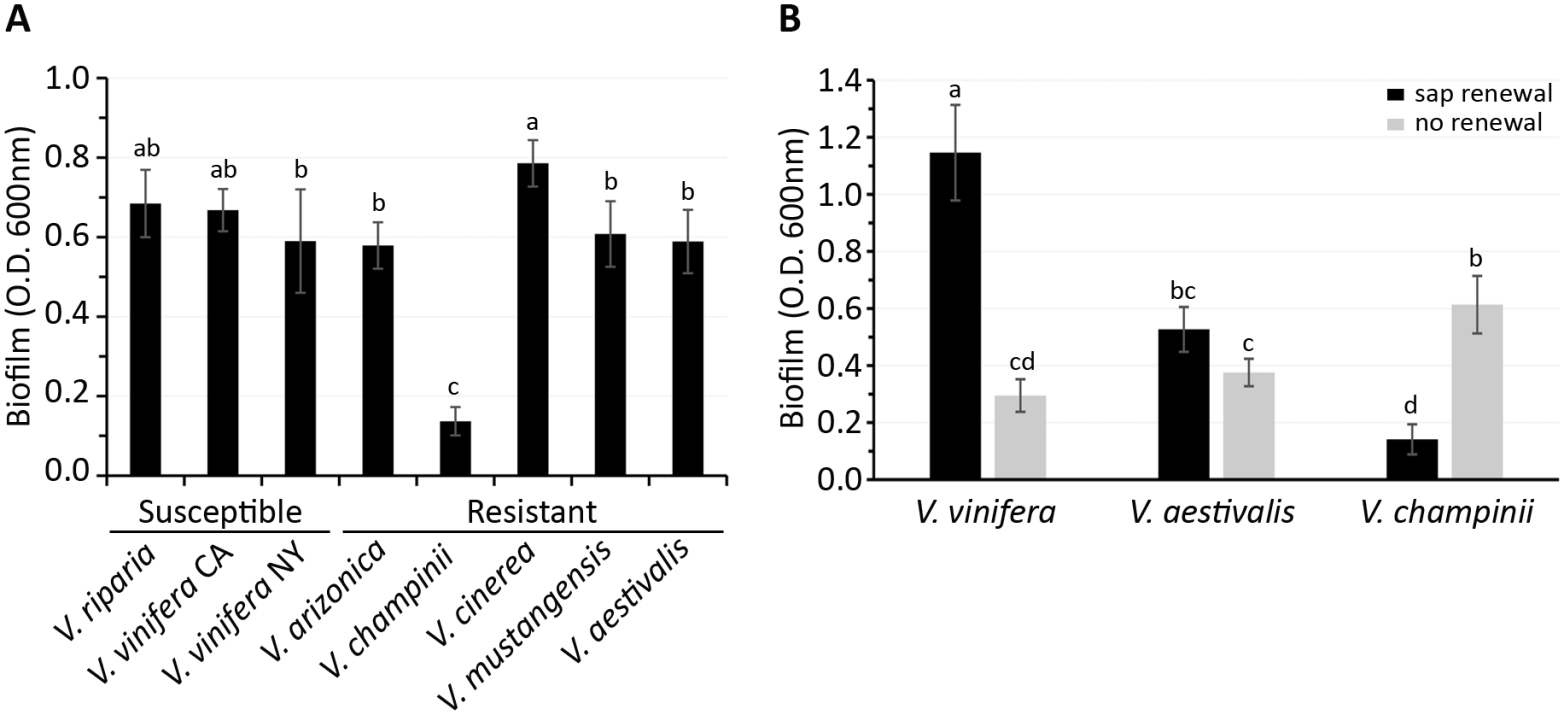
Quantification of biofilm formation by *Xylella fastidiosa* in closed or renewed sap cultures. (A) Total biofilm quantified by the crystal violet method after ten days of incubation in closed culture in 96-well plates with saps from different *Vitis* spp. Letters represent different groups as classified by Tukey’s HSD (honest significant difference) test with *p* < 0.01. (B) Biofilm levels after eight days post-inoculation in renewed (solid black bars) and closed cultures (grey bars) in glass tubes. Shown are mean biofilm quantifications and the error bars are standard deviations. Different letters represented significant differences with a *p* < 0.01.

As an initial screen, we assessed biofilm experiments when cells were in closed culture conditions in 96-well plates. The results indicated that saps vary with regard to biofilm formation and one of the resistant saps, *V*. *champinii* sap, supported less biofilm formation than other saps (Fig 1A). When compared as two groups, no clear difference was observed between biofilm formation in saps from the susceptible and resistant spp. (*p* > 0.1), when including or excluding *V*. *champinii* in the analysis.

To exclude the possibly that nutrient depletion might have an effect in the observed phenotypes, we then chose saps from *V*. *vinifera*, *V*. *aestivalis*, and *V*. *champinii* as the representatives of PD-susceptible and resistant spp., respectively, and examined biofilm formation when saps were renewed every two days in glass tubes. Results were compared to biofilm formation in the same saps in glass tubes under closed culture. For the closed culture, *X*. *fastidiosa* formed similar levels of biofilm in *V*. *vinifera* and *V*. *aestivalis* (*p* > 0.1) and more biofilm in *V*. *champinii* (*p* < 0.01) eight days post-inoculation (Fig 1B). In contrast, when sap was renewed, biofilm in *V*. *vinifera* sap was more than twice as great as that in *V*. *aestivalis* (*p* < 0.001), and more than seven times greater than that of *V*. *champinii* (*p* < 0.001). When the two culturing conditions were compared within each sap, significantly more biofilm was formed in the renewed as compared to closed culture in *V*. *vinifera* (*p* < 0.001), whereas the biofilm remained unchanged between the two culture systems (p = 0.01) in *V*. *aestivalis* and was reduced when sap was renewed compared to closed culture for *V*. *champinii* (*p* < 0.001). Taken together, data in Fig 1B shows that: 1) sap renewal culture has a significant impact on *X*. *fastidiosa* biofilm formation and 2) biofilm formation differences in saps from PD-susceptible *V*. *vinifera* versus resistant *V*. *aestivalis* and *V*. *champinii* are most pronounced in sap renewed culture.

### Comparison of *X*. *fastidiosa* population dynamics in different saps with sap renewal

After initial entry into the xylem *X*. *fastidiosa* replicates and spreads, and once a threshold population level is reached, cells switch from a motile to a sessile state and form aggregates and biofilms, leading to the clogging of xylem vessels and disease [6]. Therefore, *X*. *fastidiosa* virulence-associated behaviors, such as biofilm formation, can be regulated in a cell density-dependent manner (i.e. via quorum-sensing) [32], and thus bacterial growth is a critical step preceding the formation of biofilm. As *X*. *fastidiosa* formed significantly more biofilm in *V*. *vinifera* as compared to *V*. *aestivalis* or *V*. *champinii* in sap renewal cultures, we hypothesized that bacterial growth would be greater in *V*. *vinifera* sap.

**Fig 2:**
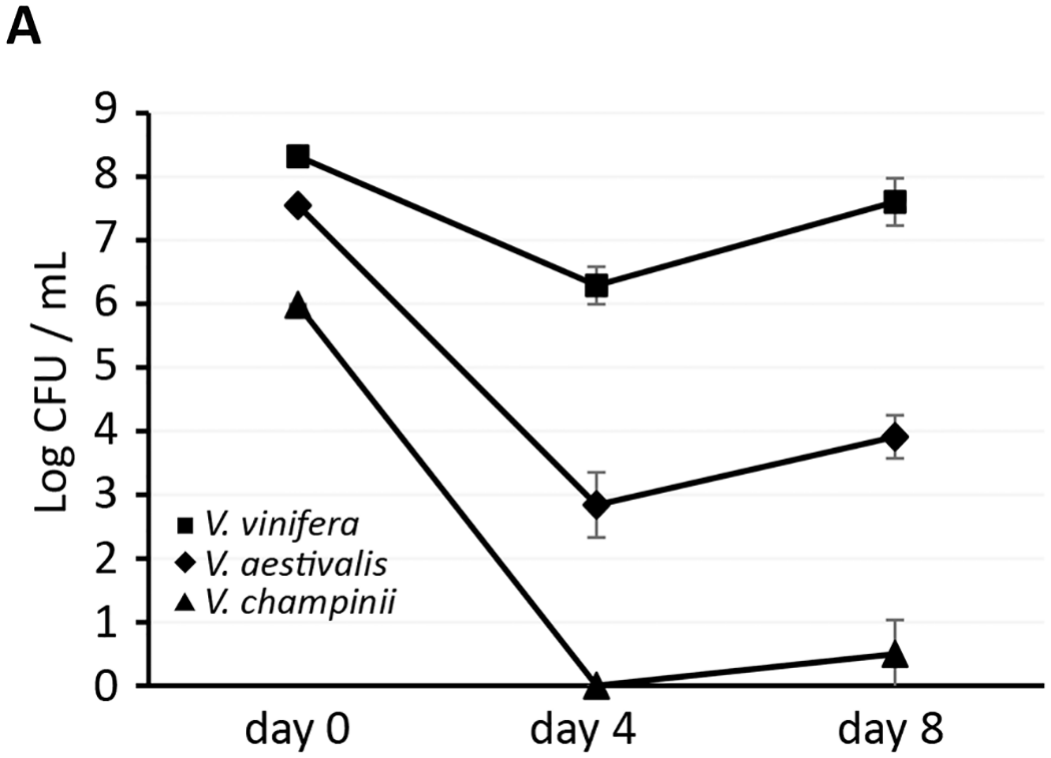
*Xylella fastidiosa* populations in different saps in renewed culture. Saps were *Vitis vinifera* (squares), *V*. *aestivalis* (diamonds), and *V*. *champinii* (triangles). Shown are mean log units of populations with standard deviation error bars.

To assess growth, *X*. *fastidiosa* cells were grown in the same way as they were for biofilm experiments shown in Fig 1B and population dynamics were determined by serial dilution plating at day zero, four, and eight. From day zero to day four the average populations declined in all three saps (Fig 2). However, the level of reduction in *V*. *vinifera* (~ 2.0 log units) sap was significantly less than those in *V*. *aestivalis* (~ 4.7 log units, *p* < 0.001) and *V*. *champinii* saps (~ 6.0 log units, *p* < 0.001). From day four to day eight, although populations in *V*. *vinifera* and *V*. *aestivalis* saps both increased, the average population level in *V*. *vinifera* was significantly higher by day eight (~ 3.5 log units) than that in *V*. *aestivalis* (*p* < 0.01). *X*. *fastidiosa* remained undetectable in *V*. *champinii* sap at both days four and eight. These data indicate that by growing *X*. *fastidiosa* in sap renewal culture a significantly greater population is achieved when cells are in *V*. *vinifera* sap as compared to saps from both resistant *Vitis* spp., and also that sap from *V*. *champinii* has an inhibitory effect on *X*. *fastidiosa* growth.

### Sap culture in microfluidic chambers to study *X*. *fastidiosa* biofilm formation and population dynamics

**Fig 3:**
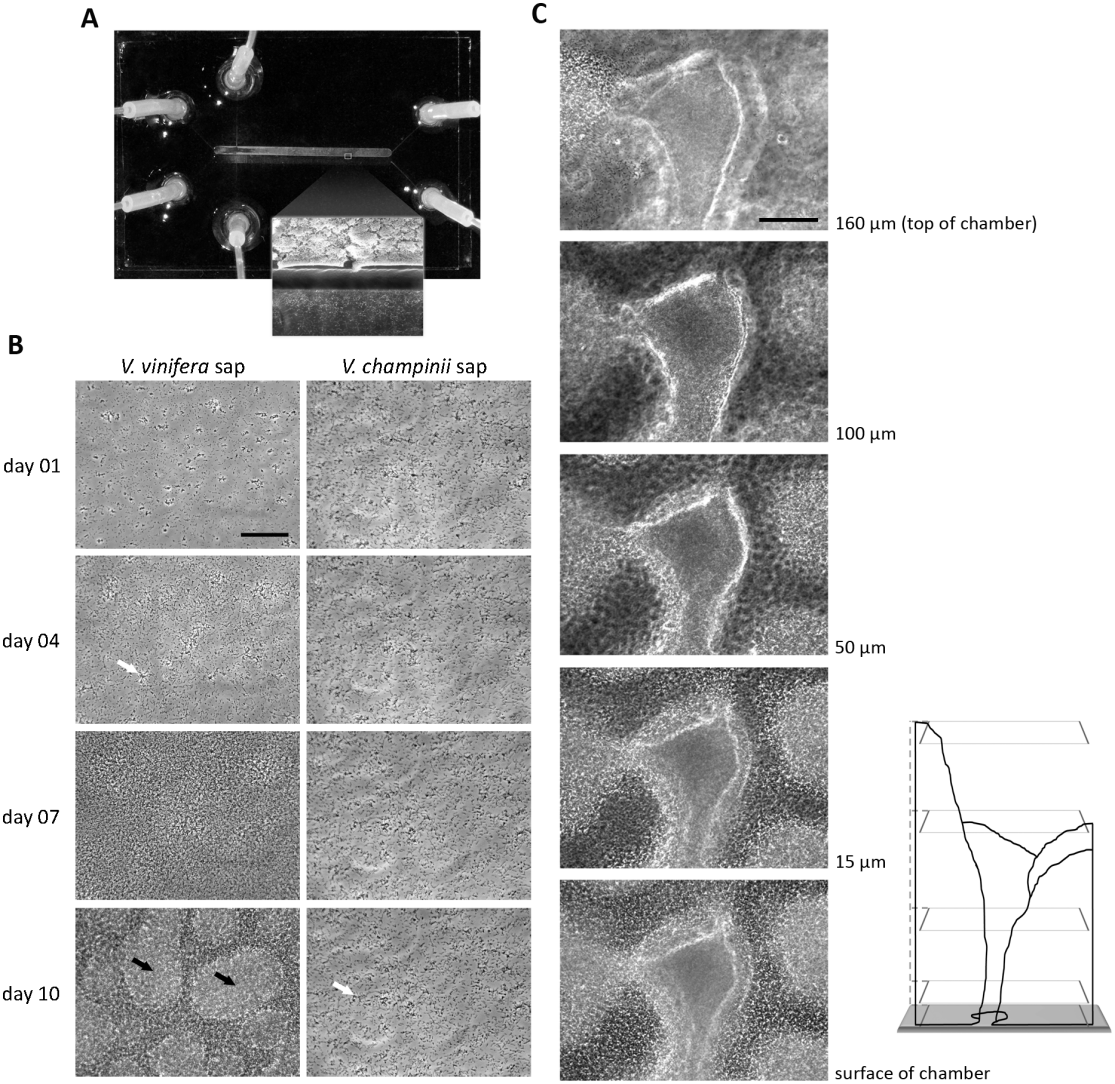
Biofilm formation by *Xylella fastidiosa* in microfluidic chambers. (A) Sap was pumped in through the inlets on the left, cells through the inlets on top and bottom, and outflow through the outlets on the right. The chamber model used has two 1 mm wide channels running in parallel in which *X*. *fastidiosa* was cultured in sap from a susceptible and resistant hosts (top and bottom channels, respectively). The insert shows scanning electron micrograph of the mature biofilm structure obtained following disassembly of the microfluidic chamber after 10 days. The wall dividing the channels is 50 μm wide. (B) Time-lapse of aggregation and biofilm development of *X*. *fastidiosa* cultured for 10 days in xylem sap from Pierce’s disease-susceptible *Vitis vinifera* (left) and Pierce’s disease-resistant *V*. *champinii* (right). White arrows point to star-shaped aggregates and black arrows shows mounds. Scale bar is 50 μm. (C) Z-scan of pillars and mound structures in mature biofilm of *X*. *fastidiosa* formed in *V*. *vinifera* sap. Series of images captured after ten days of growth under continuous flow of Pierce’s disease-susceptible *V*. *vinifera* xylem sap in a microfluidic chamber. Images obtained in a central region of the chamber and through Z-scan from the lower to upper chamber surface, which is a distance of 160 μm. Note the central region is devoid of cells and is surrounded by three major mounds (one on the left and two on the right). Scale bar is 100 μm. Diagram on bottom right represents three biofilm mounds in the Z-scan with each plane, shown as a box outlined in light grey, representing the position of the captured images.

Since sap renewal culture had a significant effect on bacterial population dynamics and biofilm formation, we also examined culturing with continuous renewal in microfluidic chambers (Fig 3A). This technology was previously used in our laboratory to study *X*. *fastidiosa* aggregation and twitching motility behaviors [33, 34]. Flow conditions in the chambers seek to mimic the plant xylem environment, in which sap is actively moving for water and nutrient transport. Culturing in chambers provides continuous fresh sap and allows for removal of bacterial cell waste and potential chemical inhibitors. It also allows for continuous observation of single cells and aggregates as well as side by side comparison of cellular behaviors in multiple saps by time-lapse imaging (Fig 3B).

After inoculation into *V*. *vinifera* sap, individual *X*. *fastidiosa* cells were observed attaching to the chamber wall surfaces and some cell aggregates were visible on day one (Fig 3B). By day four additional aggregates formed, frequently with radiating or star-shaped clusters of cells. By day seven, a lawn of cells covered the chamber surfaces and by day ten, a mature 3-D biofilm was evident. In contrast, in *V*. *champinii* sap although the initial cells attached to the chamber surfaces on day one, they stopped dividing, formed numerous small star-shaped aggregates (rod-shaped cells joined together at one cell pole), and were unable to form large aggregates or a lawn of cells at day ten. This result was consistent with the renewal growth and biofilm results and shows that direct acclimation in *V*. *champinii* or to *V*. *vinifera* prior to *V*. *champinii* (as described in Materials and Methods) had no impact. When the mature biofilm in *V*. *vinifera* was further examined in the Z-axis (orthogonal to chamber surface to which cells initially attached), three-dimensional structures such as pillars and mounds were observed, and some of these structures extended the entire height (160 μm) of the channel (Fig 3C).

**Fig 4:**
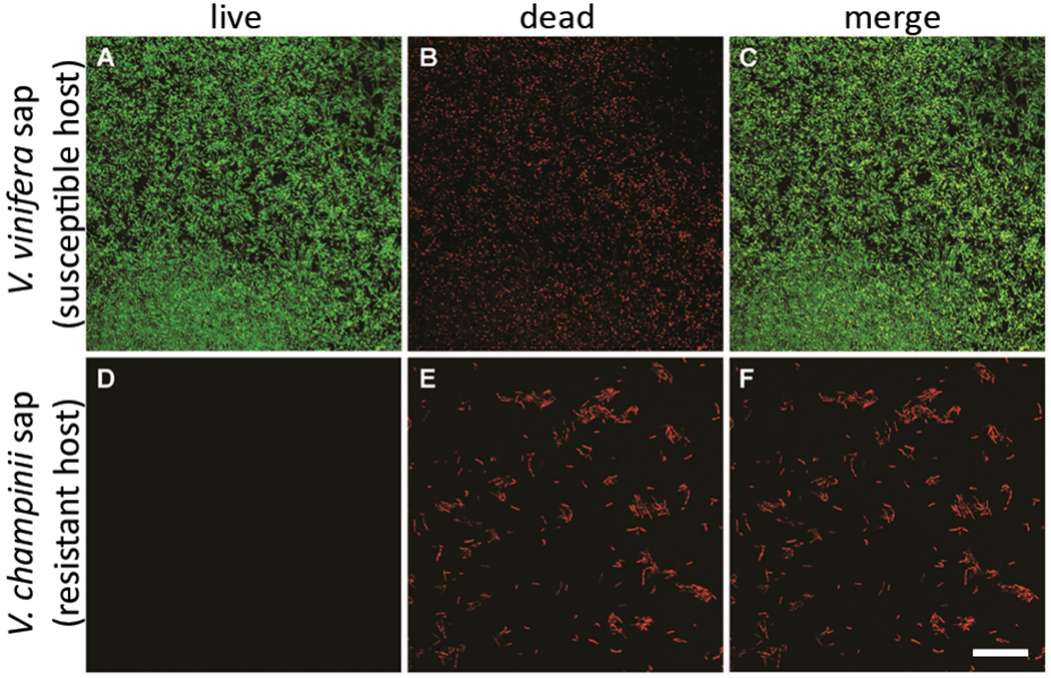
Effect of sap from resistant and susceptible *Vitis* spp. on cell viability in microfluidic chambers. eGFP-expressing *Xylella fastidiosa* cells were kept in continuous flow of sap in microfluidic chambers, and after four days the inflow syringe was replaced with the same type of sap containing PI (see Materials and Methods for more details) and continuous flow was resumed and maintained for six more days after which images where captured (after 10 days of total culturing time) by confocal laser scanning microscopy. Scale bar is 20 μm.

We also examined viability of the cells in the microfluidic chamber using the *X*. *fastidiosa* KLN59.2 strain expressing eGFP. Four days after cells were inoculated into the chamber, the inflow syringe was replaced with one containing the same sap plus PI, which only stains dead cells. At ten days post-inoculation, fluorescent images were captured and cells cultured in *V*. *vinifera* were mostly alive, as shown by the abundant living cells expressing eGFP and fewer dead cells stained by PI (Fig 4), which appear yellow or red in the merged images. In contrast, no viable cells were observed in the *V*. *champinii* sap and they also showed no indication of eGFP fluorescence. These results are consistent with those from the sap renewal culture in that *X*. *fastidiosa* formed significantly more biofilm (Fig 1B) and maintained higher viable cell numbers (Fig 2) in *V*. *vinifera* as compared to *V*. *champinii*.

### *X*. *fastidiosa* biofilm formation in saps of different *Vitis* spp. may be associated with susceptibility to PD

**Fig 5:**
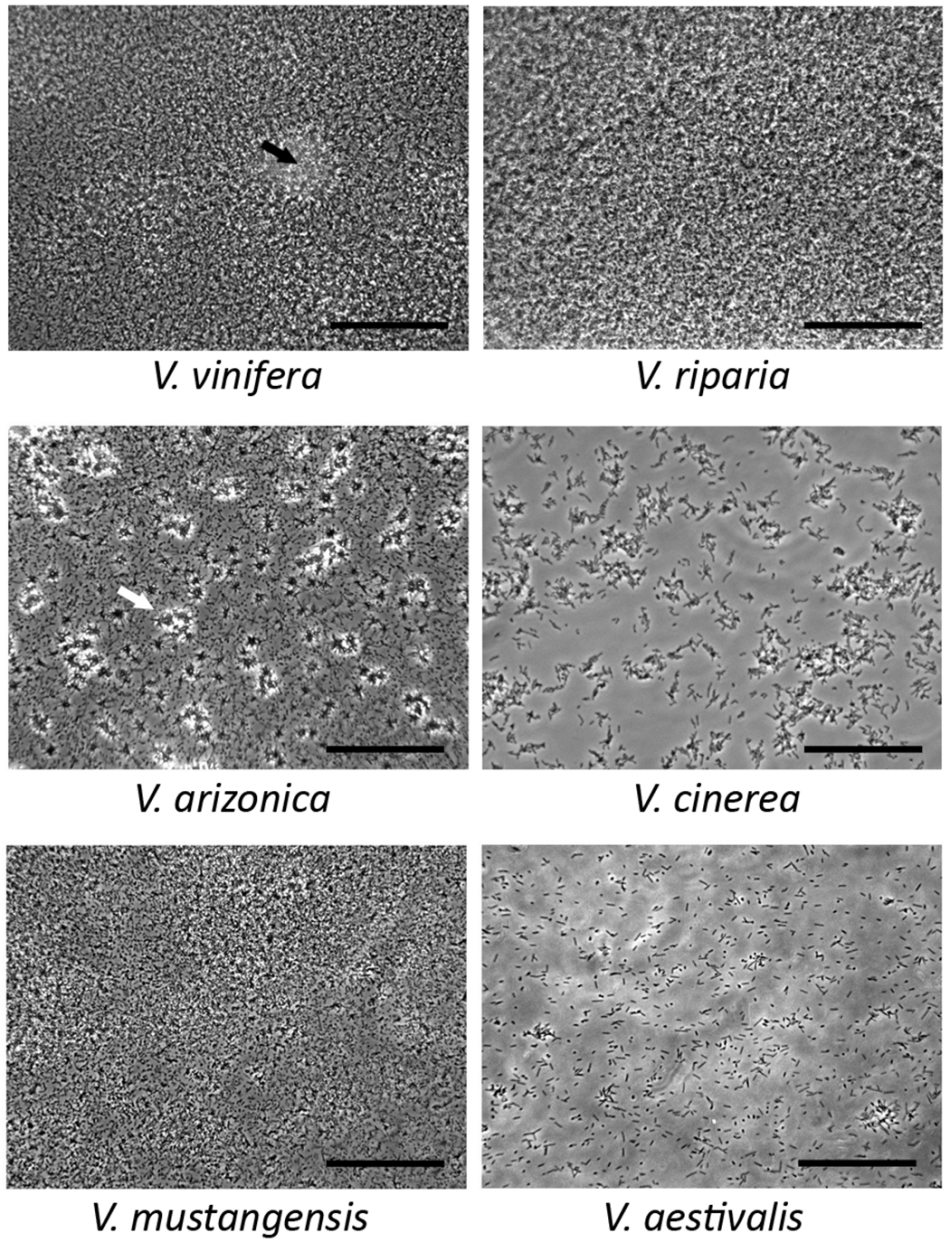
*Xylella fastidiosa* biofilm formation and aggregation in saps from PD susceptible and resistant *Vitis* spp. in microfluidic chambers. *X*. *fastidiosa* was cultured in xylem saps from different susceptible and resistant *Vitis* spp. for a period of ten days. *V*. *vinifera* was from California. Black arrow points to biofilm mound. Scale bar is 50 μm.

Based on the observed behavioral differences of *X*. *fastidiosa* in *V*. *vinifera* and *V*. *champinii* saps in microfluidic chambers, we hypothesized that biofilm formation is a general characteristic that differentiates saps from susceptible and resistant *Vitis* spp. Therefore, we examined biofilm formation in saps from two additional susceptible and four resistant *Vitis* spp. to test this hypothesis. *X*. *fastidiosa* in saps from PD-resistant spp. formed small aggregates (*V*. *cinerea* and *V*. *aestivalis*) or star-shaped aggregates (*V*. *arizonica*) ten days post-inoculation (Fig 5). *V*. *mustangensis*, was the exception in that it supported moderate amounts of biofilm and exhibited a thick cell lawn by day ten, similar to those observed in *V*. *vinifera* at day seven in Fig 3B. However, three-dimensional structures such as pillars and mounds were not observed in *V*. *mustangensis*, suggesting a structural difference as compared to the mature biofilm formed in *V*. *vinifera*. Overall, *X*. *fastidiosa* was qualitatively observed to produce thick biofilms in saps from both PD-susceptible spp. and little to no biofilm in saps from four of five of the PD-resistant spp. tested (Table 1). Interestingly, observed behaviors of *X*. *fastidiosa* in *V*. *vinifera* Chardonnay saps collected from California and New York vines did not differ; both supported high populations and strong biofilm formation in the chambers.

**Table 1:** *X*. *fastidiosa* biofilm formation in different grape saps under continuous flow in microfluidic chambers.

Xylem sap source	Biofilm	PD^a^ development	References
*V*. *riparia*	++^b^	Susceptible	[7]
*V*. *vinifera* Chardonnay NY^c^	++	Susceptible	[16]
*V*. *vinifera* Chardonnay CA	++	Susceptible	[7, 35]
*V*. *arizonica*	-	Resistant	[7, 10]
*V*. *champinii* (Ramsey or Salt Creek)	-	Resistant	[7, 12]
*V*. *cinerea* var. *helleri*	-	Resistant	[13]
*V*. *mustangensis*	+	Resistant	[7, 11]
*V*. *aestivalis* (Blue Lake)	-	Resistant	[8]

^a^ PD, Pierce’s disease.

^b^ Qualitative biofilm rating with a scale rating from “–” (no biofilm) to “++” (most biofilm production). “++”: thick lawns of cells with three-dimensional (3-D) biofilm structures such as pillars and mounds; “+”: thick lawns of cells without 3-D biofilm structures; “-”: only cell aggregates but no biofilm.

^c^ CA, California; NY, New York.

## Discussion

Research employing sap as the sole nutrient source for bacterial pathogens have highlighted its importance for examining pathogen virulence-associated behaviors that may occur *in planta* [27, 36, 37]. For example, compared to minimal or rich media, cabbage xylem sap induced specific transcriptional programming in *Xanthomonas campestris* pv. *campestris*, such as upregulation of genes involved in nutrient acquisition, detoxification, motility, and adhesion that are important during the early infection process. Moreover, biological studies with plant sap/exudates revealed the metabolic requirements for microorganisms to proliferate in different plant niches [38–40]. For example, *Salmonella enterica* requires siderophore biosynthesis to replicate and colonize alfalfa roots and lettuce leaves, both of which are iron depleted environments for the bacterium; *X*. *campestris* pv. *campestris* was found to utilize plant derived *N*-acetylglucosamine as a substrate during growth in cabbage xylem sap as well as during plant infection; and *Ralstonia solanacearum* depletes oxygen in tomato xylem, resulting in low oxygen level in xylem sap but can respire using nitrate, which is abundant in sap during plant colonization. Therefore, in this research we utilized grape sap as a natural medium for culturing *X*. *fastidiosa*.

Studying behaviors of microorganisms in natural environments can be challenging. *In vitro* systems often need to be developed to allow efficiency to carry out multiple replications while mimicking as closely as possible the environment in which the organism lives. Indeed, an increasing number of studies have used more physiologically relevant environments such as chemostat systems and flowcells to study bacterial biofilm formation [41–43]. We previously developed and employed microfluidic chambers with artificial media to gain insight into *X*. *fastidiosa* motility and aggregation behaviors in xylem vessels [33, 34]. Here we incorporated this technology with the sap medium to further create a natural environment for studying this bacterium.

Culture conditions are known to impact bacterial cell morphology and biofilm formation [44, 45]. For example, *Pseudomonas fluorescens* formed less biofilm without medium renewal than with renewal, and medium renewal affected lipopolysaccharide modification of *Escherichia coli* cells in biofilms that resulted in reduced lipid A palmitoylation. To improve our understanding of how sap chemistry affects *X*. *fastidiosa* behaviors that are associated with disease development, we compared three culture systems: closed, sap renewal, and microfluidic chambers using saps from different *Vitis* spp. Major differences in biofilm formation and cell growth were discovered, which were affected by sap origin as well as culture system. For example, no difference in biofilm formation was found between *X*. *fastidiosa* grown in *V*. *vinifera* or *V*. *aestivalis* saps in the closed cultures (Fig 1B). With sap renewal, significantly more biofilm developed in the susceptible but not in the resistant saps. This phenomenon was further confirmed in the microfluidic chambers, where *X*. *fastidiosa* formed thick lawns of biofilm in all susceptible saps but only small aggregates of various shapes in four of five of the resistant saps tested (Fig 5). Biofilm formation in *V*. *champinii* sap was less in polystyrene plates (Fig 1A) but more in glass tubes (Fig 1B) when compared to cells in saps from *V*. *vinifera* or *V*. *aestivalis*. This could due to many factors associated with the two environments, such as differences in surface materials [36, 46] or aeration [47]. Moreover, the sap chemistry of *V*. *champinii* appears unique in that it suppressed both biofilm formation and cell proliferation, which would be interesting for future research.

*X*. *fastidiosa* populations declined in all three saps (*V*. *vinifera*, *V*. *aestivalis*, and *V*. *champinii*) during the initial stage even with culture renewal (Fig 2). This could due to several possibilities. For example, immediately prior to adding cells to 100% sap they were grown in 90% and therefore possibly required acclimation to the new medium. It is also possible that because sap is nutrient poor [48], fresh sap needed to be provided more frequently than every two days. With sap renewal culture, cells acclimated in *V*. *vinifera* sap developed greater populations than acclimated cells in *V*. *aestivalis* or *V*. *champinii* saps eight days after inoculation. This result was also observed, and even more pronounced, in the microfluidic chambers where no viable cells were observed in *V*. *champinii* after ten days of culturing while most cells in *V*. *vinifera* were alive. Thus when the differences in growth and biofilm formation are compared under different culturing conditions, renewing the sap medium (whether in tubes or in chambers) is critical for determining *X*. *fastidiosa* behaviors in different grape saps that might not be seen in closed culture.

Overall, the differences in biofilm formation between susceptible and resistant saps seem to be well explained by cell growth, that is, susceptible sap supports more growth and biofilm than the saps from resistant species, at least for the three saps tested in the sap replenishment assay. Beyond the need of a threshold population size to initiate the development of a 3-D biofilm structure, other factors such as nutrient source and cation concentration also impact biofilm structure development. For example, in *Pseudomonas aeruginosa*, carbon sources impact biofilm structures with amino acids or citrates promoting formation of flat, uniform biofilms, while glucose promotes formation of large cell aggregates in structured biofilms [49]; Mg^2+^ increases *P*. *fluorescens* initial attachment to surface and also changes subsequent biofilm development and structure [50]. Further characterization and comparison of xylem sap compositions in saps from susceptible and resistant hosts may help to identify and characterize plant factors that regulate bacterial virulence behaviors such as biofilm formation.

While saps from PD-susceptible and -resistant spp. fall into two separate categories, our findings suggest that within saps from PD-resistant spp. there is a complex spectrum of responses, as observed for the different growth and biofilm formation supported by saps from *V*. *aestivalis* and *V*. *champinii*. Fritschi *et al*. classified *V*. *champinii* and *V*. *aestivalis* as belonging to separate groups based on *X*. *fastidiosa* cell population dynamics *in planta*, as cells in *V*. *champinii* spiked in population but leveled in growth while growth was more supported in *V*. *aestivalis* over time [11]. In addition, we observed that cells grown in saps from the various PD-resistant spp. in microfluidic chambers produced different aggregation phenotypes. We also observed that sap from *V*. *mustangensis*, a PD-resistant spp., supports the formation of a thick lawn of cells in the chambers. However, the cells failed to form the 3-D structures such as pillars and mounds typically seen in a mature biofilm formed in the saps from susceptible spp. such as *V*. *vinifera*. Interestingly *V*. *mustangensis* is a known reservoir for *X*. *fastidiosa* that supports cell growth [11, 51], indicating that factors other than lack of biofilm are involved in disease resistance. Aligning with our findings, a spectrum of resistance to PD is found in field studies [9, 11]. Further research, such as expression analysis of aggregation and biofilm formation associated genes of *X*. *fastidiosa* in different saps or biochemical analysis of sap components, will help to better understand the difference among different resistant spp.

Our findings are consistent with a model whereby *X*. *fastidiosa* reaches a threshold population to initiate quorum-sensing regulation resulting in formation of biofilms that can clog xylem vessels and contribute to PD [6, 52]. How this process differs between the PD-susceptible and -resistant plants is suggested by our results; we find that saps from PD-resistant species such as *V*. *champinii* and *V*. *aestivalis* do not support viable populations equivalent to PD-susceptible *V*. *vinifera* sap. Reduced populations of viable cells may fail to reach necessary threshold levels to trigger quorum sensing regulated responses, including biofilm formation [53, 54]. Reduced cell viability may be associated with low nutrient status or the presence of toxic compounds; however, additional research is needed to further understand this phenomenon. Basha *et al*. found that sap from PD-resistant *V*. *rotundifolia* is both less nutritious than *V*. *vinifera* sap and contains compounds involved in host defense against *X*. *fastidiosa* [26], indicating both nutritional and other chemical factors may be involved. Identifying the component(s) in sap that lead to these observed phenotypes in renewal culture could lead to new approaches for controlling PD.

## References

[1] Hopkins DL, Purcell AH. *Xylella fastidiosa*: cause of Pierce’s disease of grapevine and other emergent diseases. Plant Dis. 2002;86:1056–66. 10.1094/PDIS.2002.86.10.1056 30818496

[2] Saponari M, Boscia D, Nigro F, Martelli GP. Identification of DNA sequenes related to *Xylella fastidiosa* in oleander, almond and olive trees exhibiting leaf scorch symptoms in Apulia (southern Italy). J Plant Pathol. 2013;95(3):668.

[3] Zhang S, Chakrabarty PK, Fleites LA, Rayside PA, Hopkins DL, Gabriel DW. Three new Pierce's disease pathogenicity effectors identified using *Xylella fastidiosa* biocontrol strain EB92-1. PloS one. 2015;10(7):e0133796 10.1371/journal.pone.0133796 26218423 PMC4517913

[4] Nascimento R, Gouran H, Chakraborty S, Gillespie HW, Almeida-Souza HO, Tu A, et al The type II secreted lipase/esterase LesA is a key virulence factor required for *Xylella fastidiosa* pathogenesis in grapevines. Sci Rep. 2016;6:18598 10.1038/srep18598 26753904 PMC4709584

[5] Lee MW, Tan CC, Rogers EE, Stenger DC. Toxin-antitoxin systems *mqs*R/*ygi*T and *din*J/*rel*E of *Xylella fastidiosa*. Physiol Mol Plant Path. 2014;87:59–68.

[6] Chatterjee S, Almeida RP, Lindow S. Living in two worlds: the plant and insect lifestyles of *Xylella fastidiosa*. Annu Rev Phytopathol. 2008;46:243–71. 10.1146/annurev.phyto.45.062806.094342 18422428

[7] Loomis NH. Performance of *Vitis* species in the south as an indication of their relative resistance to Pierce's disease. Plant Dis Rep. 1958;42(7):833–6.

[8] Stover LH. Progress in the development of grape varieties for Florida. Proc Fla State Hort Soc. 1960;73:320–3.

[9] Krivanek AF, Walker MA. *Vitis* resistance to Pierce's disease is characterized by differential *Xylella fastidiosa* populations in stems and leaves. Phytopathology. 2005;95(1):44–52. 10.1094/PHYTO-95-0044 18943835

[10] Ruel JJ, Walker MA. Resistance to Pierce’s disease in *Muscadinia rotundifolia* and other native grape species. Am J Enol Vitic. 2006;57(2):158–65.

[11] Fritschi FB, Lin H, Walker MA. *Xylella fastidiosa* population dynamics in grapevine genotypes differing in susceptibility to Pierce's disease. Am J Encol Vitic. 2007;58(3):326–32.

[12] Lu J, Ren ZB, Cousins P. Evaluation of grape rootstocks for resistance to Pierce’s disease and adaptation to north Florida environment. Acta Hort. 2008;772:257–62.

[13] Black M. Most not wanted plants can be infected with the Pierce's disease bacterium in Texas. Texas PD Research Symposium; Driftwood, TX 2009.

[14] Fry SM, Milholland RD. Response of resistant, tolerant, and susceptible grapevine tissues to invasion by the Pierce’s disease bacterium, *Xylella fastidiosa*. Phytopathology. 1990;80:66–9.

[15] Baccari C, Lindow SE. Assessment of the process of movement of *Xylella fastidiosa* within susceptible and resistant grape cultivars. Phytopathology. 2011;101(1):77–84. 10.1094/PHYTO-04-10-0104 20822432

[16] Cursino L, Li Y, Zaini PA, De La Fuente L, Hoch HC, Burr TJ. Twitching motility and biofilm formation are associated with *tonB1* in *Xylella fastidiosa*. FEMS Microbiol Lett. 2009;299(2):193–9. 10.1111/j.1574-6968.2009.01747.x 19735464

[17] Shi XY, Dumenyo CK, Hernandez-Martinez R, Azad H, Cooksey DA. Characterization of regulatory pathways in *Xylella fastidiosa*: genes and phenotypes controlled by *gacA*. Appl Environ Microbiol. 2009;75(8):2275–83. 10.1128/AEM.01964-08 19218414 PMC2675201

[18] Cursino L, Galvani CD, Athinuwat D, Zaini PA, Li Y, De La Fuente L, et al Identification of an operon, Pil-Chp, that controls twitching motility and virulence in *Xylella fastidiosa*. Mol Plant Microbe Interact. 2011;24(10):1198–206. 10.1094/MPMI-10-10-0252 21692637

[19] Matsumoto A, Huston SL, Killiny N, Igo MM. XatA, an AT-1 autotransporter important for the virulence of *Xylella fastidiosa* Temecula 1. Microbiologyopen. 2012;1(1):33–45. 10.1002/mbo3.6 22950010 PMC3426408

[20] Killiny N, Martinez RH, Dumenyo CK, Cooksey DA, Almeida RP. The exopolysaccharide of *Xylella fastidiosa* is essential for biofilm formation, plant virulence, and vector transmission. Mol Plant Microbe Interact. 2013;26(9):1044–53. 10.1094/MPMI-09-12-0211-R 23678891

[21] Cursino L, Athinuwat D, Patel KR, Galvani CD, Zaini PA, Li Y, et al Characterization of the *Xylella fastidiosa* PD1671 gene encoding degenerate c-di-GMP GGDEF/EAL domains, and its role in the development of Pierce's disease. PloS one. 2015;10(3):e0121851 10.1371/journal.pone.0121851 25811864 PMC4374697

[22] de Souza AA, Ionescu M, Baccari C, da Silva AM, Lindow SE. Phenotype overlap in *Xylella fastidiosa* is controlled by the cyclic di-GMP phosphodiesterase Eal in response to antibiotic exposure and diffusible signal factor-mediated cell-cell signaling. Appl Environ Microbiol. 2013;79(11):3444–54. 10.1128/AEM.03834-12 23542613 PMC3648042

[23] Chatterjee S, Killiny N, Almeida RP, Lindow SE. Role of cyclic di-GMP in *Xylella fastidiosa* biofilm formation, plant virulence, and insect transmission. Mol Plant Microbe Interact: MPMI. 2010;23(10):1356–63. 10.1094/MPMI-03-10-0057 20831412

[24] Andersen PC, Brodbeck BV, Oden S, Shriner A, Leite B. Influence of xylem fluid chemistry on planktonic growth, biofilm formation and aggregation of *Xylella fastidiosa*. FEMS Microbiol Lett. 2007;274(2):210–7. 17610515 10.1111/j.1574-6968.2007.00827.x

[25] Cheng DW, Lin H, Walker MA, Stenger DC, Civerolo EL. Effects of grape xylem sap and cell wall constituents on in vitro growth, biofilm formation and cellular aggregation of *Xylella fastidiosa*. Eur J Plant Pathol. 2009;125:213–22.

[26] Basha SM, Mazhar H, Vasanthaiah HK. Proteomics approach to identify unique xylem sap proteins in Pierce's disease-tolerant *Vitis* species. Appl Biochem Biotechnol. 2010;160(3):932–44. 10.1007/s12010-009-8620-1 19412582

[27] Zaini PA, De La Fuente L, Hoch HC, Burr TJ. Grapevine xylem sap enhances biofilm development by *Xylella fastidiosa*. FEMS Microbiol Lett. 2009;295(1):129–34. 10.1111/j.1574-6968.2009.01597.x 19473259

[28] Davis MJ, French WJ, Schaad NW. Axenic culture of the bacteria associated with phony disease of peach and plum leaf scald. Curr Microbiol. 1981;6:309–14.

[29] Newman KL, Almeida RP, Purcell AH, Lindow SE. Use of a green fluorescent strain for analysis of *Xylella fastidiosa* colonization of *Vitis vinifera*. Appl Environ Microbiol. 2003;69(12):7319–27. 14660381 10.1128/AEM.69.12.7319-7327.2003 PMC310014

[30] Minsavage GV, Thompson CM, Hopkins DL, Leite RMCBC, Stall RE. Development of a polymerase chain reaction protocol for detection of *Xylella fastidiosa* in plant tissue. Phytopathology. 1994;84:456–61.

[31] Meng Y, Li Y, Galvani CD, Hao G, Turner JN, Burr TJ, et al Upstream migration of *Xylella fastidiosa* via pilus-driven twitching motility. J Bacteriol. 2005;187(16):5560–7. 16077100 10.1128/JB.187.16.5560-5567.2005 PMC1196070

[32] Chatterjee S, Wistrom C, Lindow SE. A cell-cell signaling sensor is required for virulence and insect transmission of *Xylella fastidiosa*. Proc Natl Acad Sci U S A. 2008;105(7):2670–5. 10.1073/pnas.0712236105 18268318 PMC2268194

[33] De La Fuente L, Burr TJ, Hoch HC. Autoaggregation of *Xylella fastidiosa* cells is influenced by type I and type IV pili. App Environ Microbiol. 2008;74(17):5579–82. 10.1128/AEM.00995-08 PMC2546647 18641157

[34] De La Fuente L, Burr TJ, Hoch HC. Mutations in type I and type IV pilus biosynthetic genes affect twitching motility rates in *Xylella fastidiosa*. J Bacteriol. 2007;189(20):7507–10. 17693510 10.1128/JB.00934-07 PMC2168456

[35] Raju BC, Goheen AC. Relative sensitivity of selected grapevine cultivars to Pierce's disease bacterial inoculations. Am J Enol Vitic. 1981;32(2):155–8.

[36] Shi X, Bi J, Morse JG, Toscano NC, Cooksey DA. Effect of xylem fluid from susceptible and resistant grapevines on developmental biology of *Xylella fastidiosa*. Eur J Plant Pathol. 2013;135:127–35.

[37] Duge de Bernonville T, Noel LD, SanCristobal M, Danoun S, Becker A, Soreau P, et al Transcriptional reprogramming and phenotypical changes associated with growth of *Xanthomonas campestris* pv. *campestris* in cabbage xylem sap. FEMS Microbiol Ecol. 2014;89(3):527–41. 10.1111/1574-6941.12345 24784488

[38] Boulanger A, Zischek C, Lautier M, Jamet S, Rival P, Carrere S, et al The plant pathogen *Xanthomonas campestris* pv. *campestris* exploits N-acetylglucosamine during infection. mBio. 2014;5(5):e01527–14. 10.1128/mBio.01527-14 25205095 PMC4173781

[39] Hao LY, Willis DK, Andrews-Polymenis H, McClelland M, Barak JD. Requirement of siderophore biosynthesis for plant colonization by *Salmonella enterica*. Appl Environ Microbiol. 2012;78(13):4561–70. 10.1128/AEM.07867-11 22522683 PMC3370490

[40] Dalsing BL, Truchon AN, Gonzalez-Orta ET, Milling AS, Allen C. *Ralstonia solanacearum* uses inorganic nitrogen metabolism for virulence, ATP production, and detoxification in the oxygen-limited host xylem environment. mBio. 2015;6(2):e02471 10.1128/mBio.02471-14 25784703 PMC4453514

[41] Kim J, Park HD, Chung S. Microfluidic approaches to bacterial biofilm formation. Molecules. 2012;17(8):9818–34. 10.3390/molecules17089818 22895027 PMC6268732

[42] Karimi A, Karig D, Kumar A, Ardekani AM. Interplay of physical mechanisms and biofilm processes: review of microfluidic methods. Lab Chip. 2015;15(1):23–42. 10.1039/c4lc01095g 25385289 PMC4261921

[43] Drake DR, Brodgen KA. Continuous-culture chemostat systems and flowcells as methods to investigate microbial interactions In: Brodgen KA, Guthmiller JM, editors. Polymicrobial diseases. Washington, D.C.: ASM Press; 2002. 21735561

[44] Sillankorva S, Neubauer P, Azeredo J. Isolation and characterization of a T7-like lytic phage for *Pseudomonas fluorescens*. BMC Biotechnol. 2008;8:80 10.1186/1472-6750-8-80 18954452 PMC2582237

[45] Chalabaev S, Chauhan A, Novikov A, Iyer P, Szczesny M, Beloin C, et al Biofilms formed by Gram-negative bacteria undergo increased lipid A palmitoylation, enhancing *in vivo* survival. mBio. 2014;5(4). 10.1128/mBio.01116-14 PMC4147861 25139899

[46] Li Y, Hao G, Galvani CD, Meng Y, De La Fuente L, Hoch HC, et al Type I and type IV pili of *Xylella fastidiosa* affect twitching motility, biofilm formation and cell-cell aggregation. Microbiology. 2007;153(Pt 3):719–26. 17322192 10.1099/mic.0.2006/002311-0

[47] Duetz WA, Ruedi L, Hermann R, O'Connor K, Buchs J, Witholt B. Methods for intense aeration, growth, storage, and replication of bacterial strains in microtiter plates. Appl Environ Microbiol. 2000;66(6):2641–6. 10831450 10.1128/aem.66.6.2641-2646.2000 PMC110593

[48] Bove JM, Garnier M. Phloem- and xylem-restricted plant pathogenic bacteria. Plant Sci. 2002;163:1083–98.

[49] Klausen M, Heydorn A, Ragas P, Lambertsen L, Aaes-Jorgensen A, Molin S, et al Biofilm formation by *Pseudomonas aeruginosa* wild type, flagella and type IV pili mutants. Mol Microbiol. 2003;48(6):1511–24. 12791135 10.1046/j.1365-2958.2003.03525.x

[50] Song B, Leff LG. Influence of magnesium ions on biofilm formation by Pseudomonas fluorescens. Microbiol Res. 2006;161(4):355–61. 16517137 10.1016/j.micres.2006.01.004

[51] McGaha LA, Jackson B, Bextine B, McCullough D, Morano L. Potential plant reservoirs for *Xylella fastidiosa* in south Texas. Am J Enol Vitic. 2007;58(3):398–401.

[52] Sun Q, Sun Y, Walker MA, Labavitch JM. Vascular occlusions in grapevines with Pierce's disease make disease symptom development worse. Plant Physiol. 2013;161(3):1529–41. 10.1104/pp.112.208157 23292789 PMC3585614

[53] Ham JH. Intercellular and intracellular signalling systems that globally control the expression of virulence genes in plant pathogenic bacteria. Mol Plant Pathol. 2013;14(3):308–22. 10.1111/mpp.12005 23186372 PMC6638695

[54] Danhorn T, Fuqua C. Biofilm formation by plant-associated bacteria. Ann Rev Microbiol. 2007;61:401–22. 17506679 10.1146/annurev.micro.61.080706.093316

